# Two-year follow-up of patients with post-COVID-19 condition in Sweden: a prospective cohort study

**DOI:** 10.1016/j.lanepe.2023.100595

**Published:** 2023-02-24

**Authors:** Carl Wahlgren, Gustaf Forsberg, Anestis Divanoglou, Åse Östholm Balkhed, Katarina Niward, Sören Berg, Richard Levi

**Affiliations:** a Department of Rehabilitation Medicine and Department of Health, Medicine and Caring Sciences, Linköping University, Linköping, Sweden; b Department of Cardiothoracic and Vascular Surgery and Department of Health, Medicine and Caring Sciences, Linköping University, Linköping, Sweden; c Department of Infectious Diseases and Department of Biomedical and Clinical Sciences, Linköping University, Linköping, Sweden

**Keywords:** SARS-CoV-2, Post-COVID condition, 2-Year follow-up, Residual symtoms, Rehabilitation, COVID-19, Long-term, Longitudinal

## Abstract

**Background:**

Few studies have reported the long-term health effects of COVID-19. The regional population-based Linköping COVID-19 study (LinCoS) included all patients hospitalised due to COVID-19 during the first pandemic wave. Four months post-discharge, over 40% (185/433) experienced persisting symptoms and activity/participation limitations, indicating post-COVID-19 condition (PCC). The present follow-up study aimed to determine the long-term recovery among these patients 24 months post-admission.

**Methods:**

This prospective cohort study included all patients from LinCoS with PCC at four months post-discharge. We repeated the same structured interview at a 24-month follow-up to identify persisting symptoms and their impact on daily life. Intercurrent health issues were identified by reviewing medical records.

**Findings:**

Of 185 patients with PCC at 4 months post-discharge, 181 were alive at the 24-month assessment and 165 agreed to participate. Of those, 21% (35/165) had been readmitted to hospital for various causes in the interim period. The majority of patients (139/165, 84%) reported persisting problems affecting everyday life at 24 months. Significant improvements were seen in the prevalence and magnitude of some symptoms/limitations compared with four months post-discharge. Cognitive, sensorimotor, and fatigue symptoms were the most common persisting symptoms at 24 months. No clear difference was evident between individuals treated in the intensive care unit (ICU) and non-ICU-treated individuals. Approximately half of those who were on sick leave related to PCC at four months after infection were on sick leave at 24 months.

**Interpretation:**

This is one of the first studies to report 2-year outcomes in patients with PCC following COVID-19 hospitalisation. Despite some improvements over time, we found a high prevalence of persisting symptoms and a need for long-term follow-up and rehabilitation post COVID-19 infection.

**Funding:**

The study was funded by 10.13039/100016670Region Östergötland.

**Research in context**

**Evidence before this study**

The concept of “post-COVID-19 condition” (PCC) has been established by the World Health Organization (WHO). A large variety of symptoms has been reported, including mental fatigue, affective symptoms, cognitive deficits, dyspnea, palpitations, muscle weakness and pain. There are conflicting reports regarding risk factors for persistent symptoms post-infection, with some studies indicating that disease severity and female sex increase risk. As COVID-19 is a new disease, the 2-year recovery rate for patients with PCC has yet to be determined. We performed a PubMed search with no language restrictions up to December 20th, 2022, with search terms “(COVID-19 OR SARS-CoV-2 OR Coronavirus disease 2019 OR 2019-nCoV) AND (Long COVID OR Post Covid Condition OR Post-Acute COVID-19 Syndrome OR discharge OR residual OR persisting) AND (follow-up OR two-year follow-up OR outcome)”, yielding 11,418 results. After adding filters for English language and publication after July 1st, 2022, and manual literature screening, relevant studies identified were three longitudinal cohort studies from China, a retrospective registry-based study primarily from the US, and one longitudinal cohort study from Italy of non-hospitalised patients that was primarily focused on post-infectious alterations in the sense of smell or taste. Finally, we found one cross-sectional study from Spain. To the best of our knowledge, there are no 2-year longitudinal follow-up studies of PCC from any European country.

**Added value of this study**

This is one of the first studies to report 2-year outcomes in patients with PCC who were initially hospitalised due to COVID-19. The study presents the prevalence of persisting symptoms, as well as symptom clusters and their trajectories of change over time. Although a diminishing symptom load was found, the majority of patients with symptoms at the initial follow-up after discharge still reported problems affecting their daily life two years after acute infection. Approximately half of the patients who were on sick leave due to PCC at four months after infection were still on sick leave at the 24-month follow-up.

**Implications of all the available evidence**

Results indicate that PCC following hospital-treated COVID-19, although showing improvement at 2-year follow-up, still poses a healthcare challenge in the majority of patients. Further longitudinal studies are required to corroborate these findings, including both larger cohorts and in-depth objective tests of cognitive, affective and respiratory issues at 24 months post-infection and beyond.

## Introduction

### Background

Common post-COVID-19 symptoms include dyspnea, muscle weakness, impaired fitness, mental fatigue, affective symptoms and cognitive deficits.^1^^,^^2^ Presence of persisting symptoms several months after acquiring SARS-CoV-2 infection has been denoted as “Long COVID”, “post-COVID syndrome” and, most recently, as “post-COVID-19 condition” (PCC).^3^ In comparison with influenza, the variety and prevalence of lingering symptoms seems much greater after COVID-19.^2^^,^^4^ Some reports indicate that PCC is associated with female sex, presence of significant comorbidities and increasing age.^5^, ^6^, ^7^, ^8^ A higher number of symptoms during the acute phase of COVID-19 was also associated with higher risk for PCC. However, some reports indicate that the degree of dyspnea post-hospitalisation for COVID-19 may not be related to the severity of the acute disease course.^1^^,^^9^^,^^10^ In a meta-analysis by the Global Burden of Disease Long COVID Collaborators,^8^ a total of 6.2% of survivors after symptomatic SARS-CoV-2 infection during the pandemics’ initial two years experienced symptoms of PCC three months after infection.

In the Swedish regional population-based Linköping COVID-19 study (LinCoS), patients with at least fair premorbid health status hospitalised for COVID-19 underwent a screening interview at four months post-discharge (n = 433).^1^ Although prevalence of self-reported cognitive and affective problems at follow-up was similar regardless of disease severity, limb weakness, difficulties returning to everyday activities and walking >1 km were more common in patients with more severe COVID-19.^1^ Over 40% (185/433) experienced activity/participation limitations affecting their daily life consistent with PCC, suggesting further rehabilitation needs.^1^ The latter cohort received a multi-professional clinical assessment at five months after the acute infection. Clinical assessment confirmed a broad array of deficits for respiratory, visual, auditory, motor, sensory and cognitive functions.^11^ An objective multi-domain neurocognitive test battery administered in person by a neuropsychologist further indicated a wide range of deficits in over one third of that cohort.^12^ In a subgroup of 35 patients who underwent magnetic resonance imaging (MRI) of the brain MRI, 25 (71%) had abnormalities, with multiple white matter lesions comprising the most common finding.^13^

There is currently limited understanding and evidence regarding long-term outcomes in people diagnosed with PCC. A Chinese study investigating 2-year outcomes in previously hospitalised patients reported a decrease in symptom burden, with higher risks for persisting symptoms in those with severe disease.^14^ In a large retrospective registry-based study (primarily from the US), an increased risk of psychotic disorder, cognitive deficits, dementia and epilepsy two years after initial infection was found.^15^ Another longitudinal Chinese study of lung function trajectories two years after infection identified a decrease in dyspnea as measured by the mMRC dyspnea scale.^16^ Additionally, deteriorated mental health post infection may be worsened due to lock-downs and other restrictions enforced to limit the spread of the infection,^17^ with global prevalence of anxiety and depression disorders rising in the wake of the pandemic.^18^ To the best of our knowledge, there are no 2-year follow-up studies of PCC from any European country. Further prospective systematic evaluations of the prognosis of PCC are needed to clarify its burden on public health to adequately manage its long-term effects.

### Objectives

The present study explored persisting symptoms and activity/participation limitations at 24 months after hospital admission due to COVID-19 in a cohort of patients with symptoms consistent with PCC four months post-discharge. Specifically, we investigated the following.

1. Two-year outcomes,
2. Mortality and readmission rates in the interim period,
3. Potential correlations between prognosis and initial disease severity.

## Methods

### Study design, setting and participants

The present study is a 24-month longitudinal follow-up of the well-defined LinCoS cohort.^1^^,^^11^, ^12^, ^13^ Structured screening interviews, identical to those at a previous 4-month follow-up,^1^ were performed. Reporting is in accordance with the Strengthening the Reporting of Observational Studies in Epidemiology (STROBE) guidelines for cohort studies.

A total of 745 patients with a positive polymerase chain reaction (PCR) for SARS-CoV-2 were admitted to hospital for COVID-19 during the first pandemic wave between March 1st and May 31st, 2020, in Region Östergötland (population of approximately 450,000). This region is one of 21 Swedish healthcare regions and has three hospitals: one tertiary care university hospital with approximately 400 beds and two general hospitals with 241 and 76 beds, respectively. Thirty Intensive Care Unit (ICU) beds were available at the beginning of the pandemic, which increased to 52 during the first pandemic wave. After excluding non-COVID-19 related hospitalisations (i.e., coincidental COVID-19), in-hospital deaths, major comorbidities (e.g., dementia or terminal cancer) and dropouts, 433 individuals were screened for PCC at four months after infection. This was performed using a structured telephone interview, where 185/433 (42.7%) patients reported symptoms consistent with PCC severe enough to impair daily activities. These patients were considered eligible for the current study and thus recruitment was attempted among this sample. Interviews were performed via telephone by two of the authors (CW and GF) using a structured interview guide (described below). A third interviewer, with more experience in interpreter-mediated interviews, was used when interviews could not be performed in Swedish or English (23 cases (13.9%)).

### Ethics

The Swedish Ethical Review Authority approved the study (Dnr 2020-03029, 2020-04443 and 2021-07038). In accordance with the ethics approval, the need for a written informed consent was waived given that the follow-up procedure also formed part of a clinical follow-up.

### Variables and data sources

The interview guide comprised 37 questions, of which 25 addressed bodily functions and 12 addressed activity and participation limitations.^1^ Interviews were standardized by use of this structured interview guide, instructing each of the three interviewers how to ask and respond. To further standardize how interviewers manage unusual or deviant responses all authors met for a weekly discussion. Interviewees were instructed to only consider symptoms related to COVID-19 and, when present, grade their respective impact on everyday life on a scale of 1–5 (1: no impact; 2: minor impact; 3: moderate impact; 4: high impact; 5: very high impact). General health was assessed by participants rating their current subjective health status on a five-point Likert scale, ranging from very good to very bad, similar to the first question regarding overall health of the WHO health survey questionnaire.^19^ Dyspnea was evaluated using the modified Medical Research Council (mMRC) dyspnea scale, widely accepted for follow-up of shortness of breath post COVID-19.^20^^,^^21^ Interview questions are available as supplementary information translated from Swedish to English. Data pertaining to comorbidities and health issues during the interim period up until the 24-month follow-up were retrieved from medical records.

The research team comprised medical specialists in infectious diseases, critical care, neurology, and rehabilitation medicine. The team met regularly and discussed individual needs of medical attention as disclosed by the interviews, and provided referrals to relevant caregivers when indicated.

To facilitate comparisons between the 4- and 24-month follow-ups, symptoms were clustered into seven domains which had been identified through an explorative factor analysis of 426 interviews presented in a previous LinCoS study pertaining to the 4-month assessment.^22^ These domains comprise symptoms related to vision (Domain I), sensorimotor dysfunction (Domain II), cognition (Domain III), affective symptoms (Domain IV), swallowing (Domain V), voice (Domain VI) and mental fatigue (Domain VII). Three symptoms (dizziness, hearing loss and altered smell/taste) did not fit the factor analysis, and another two (difficulty managing work/studies and experienced falls after discharge) were excluded as the first had a response rate below 50% and the second did not refer to the specific situation at the time of the interview.

Disease severity during hospitalisation for COVID-19 was classified using the highest grade achieved on the World Health Organization (WHO) Clinical Progression Scale (CPS)^23^ for the entire cohort. According to the WHO CPS, patients with grade 4 or 5 were categorized as having moderate disease severity and cases with WHO CPS 6–9 as severe. Additionally, for ICU-treated patients, the Sequential Organ Failure Assessment (SOFA)^24^ and Simplified Acute Physiology Score III (SAPS3)^25^ scores were used to determine the severity of organ failures.

### Statistics

Statistical analysis was performed using IBM SPSS vs. 27. Data are presented as means and standard deviations (SD) for normally distributed continuous variables; as medians and interquartile ranges (IQR) for non-normally distributed numeric variables; and as n (%) for categorical data. Comparisons over time for ordinal data were made using paired Wilcoxon signed-rank tests. Comparisons for normally distributed continuous variables were made using t-tests. Comparisons of occupational status (dichotomized) over time were made using McNemar's tests. No imputation was performed. Normality was assessed using Shapiro–Wilks tests. A p-value <0.05 was used to denote statistical significance throughout the paper unless otherwise noted.

### Role of the funding source

The study was funded by Region Östergötland. The funder had no role in data collection, analysis, interpretation, study design or writing of the report.

## Results

### Cohort

**Fig. 1:**
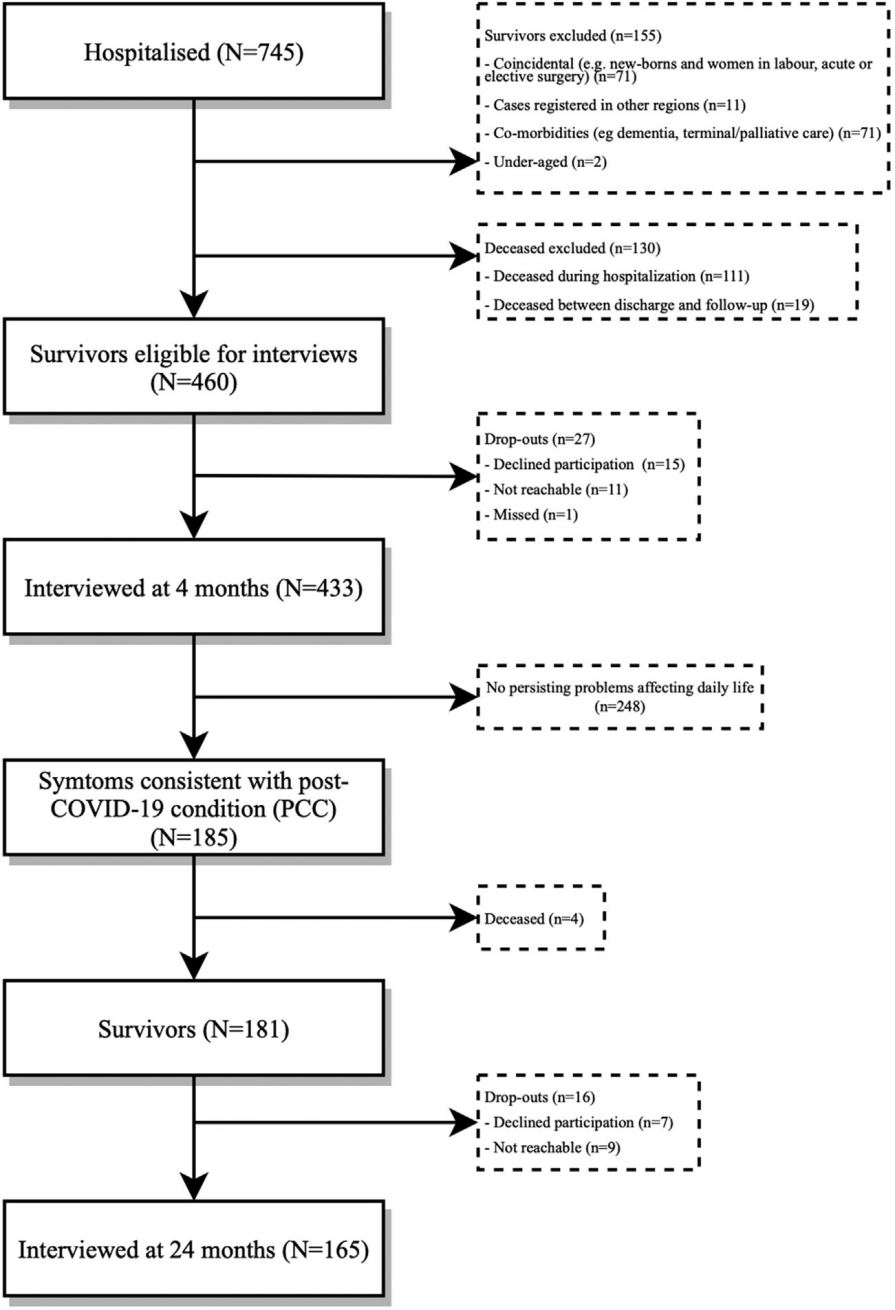
**Study Flowchart.** Study flowchart for patients hospitalised for COVID-19 during March 1st–May 31st, 2020, in Region Östergötland, Sweden. The bottom-most box represents the cohort presented in this paper.

For a detailed description of the initial disease course, as well as for the results of the 4-month follow-up, see Divanoglou et al. (2021),^1^ Hellgren et al. (2022)^22^ and Forsberg et al. (2022).^26^ Of the initial 185 eligible patients at the 4-month follow-up, four (2.2%) had died prior to the 24-month follow-up. Out of the 181 survivors, 165 (91.2%) accepted participation, seven (3.9%) declined participation and nine (5.0%) could not be contacted despite several attempts (Fig. 1). For patient characteristics of the cohort, see Table 1. Median follow-up time was 719 days after hospital admission (IQR 702–753).

**Table 1:** Patient characteristics.

Characteristics	Total (n = 165)	ICU (n = 47)	Non-ICU (n = 118)
Female	61 (40%)	9 (19%)	52 (44%)
Age	61 (SD 13)	64 (SD 11)	60 (SD 14)
Cardiovascular disease	31 (18%)	4 (9%)	27 (23%)
Hypertension	68 (39%)	20 (43%)	48 (41%)
Diabetes	38 (22%)	12 (26%)	26 (22%)
Obesity	16 (9%)	8 (17%)	8 (7%)
Chronic respiratory disease	32 (18%)	12 (26%)	20 (17%)
Chronic kidney disease	10 (6%)	6 (13%)	4 (3%)
Cancer	5 (3%)	2 (4%)	3 (3%)
Psychiatric disease	19 (11%)	5 (11%)	14 (12%)
No previous comorbidities	52 (30%)	13 (28%)	39 (33%)
Clinical Progression Scale (CPS)			
CPS 4-5	107 (65%)	1 (2%)	106 (90%)
CPS 6	16 (10%)	4 (9%)	12 (10%)
CPS 7–9	42 (25%)	42 (89%)	0 (0%)
Ethnicity			
Swedish	109 (66%)	30 (64%)	79 (67%)
Other European	19 (12%)	5 (11%)	14 (12%)
Middle Eastern/North African	28 (17%)	9 (19%)	19 (16%)
Other African	3 (2%)	2 (4%)	1 (1%)
Other	4 (2%)	1 (2%)	3 (3%)
Unknown	2 (1%)	0 (0%)	2 (2%)
Current smoker (n = 151)	12 (8%)	4 (9%)	12 (11%)
Ever smoked (n = 140)	81 (58%)	24 (75%)	57 (53%)
Vaccination status at 24-months (doses)			
0	10 (6%)	4 (9%)	6 (5%)
1	3 (2%)	0 (0%)	3 (3%)
2	27 (16%)	6 (13%)	21 (18%)
3 or more	124 (75%)	36 (77%)	88 (75%)
Unknown	1 (1%)	1 (2%)	0 (0%)

Data obtained from medical records. Comorbidities represented by categories of ICD-10 diagnostic codes. “No previous comorbidities” indicates that neither of the disease categories listed above were present.

The entire cohort comprised 104 (63.0%) males. The ICU-treated subgroup had a greater predominance of males (81%, 38/47) than the non-ICU-treated subgroup (56%, 66/118). Among the 47 included ICU patients, the median length of hospital stay was 25 days (17–45) and 43 (91%) had received invasive mechanical ventilation for a median duration of 17 days (9–22). Median SOFA and SAPS3 scores upon admission to ICU were 4 (3–4) and 52 (47–57), respectively. Among the non-ICU-treated patients, no previous comorbidities were present in 67% (79) and the median length of hospital stay was 4 (2–9) days.

Thirty-five of the 165 participants (21.2%) had been readmitted to hospital for a median of 2 (IQR 1–4) times between the 4 and 24-month follow-ups. The diagnostic categories for these readmissions were (n): Cardiovascular (19), Trauma/injuries (16), Urogenital diseases (14), Endocrine diseases (10), Infectious diseases (10, including 3 COVID-19 reinfections), Respiratory diseases/symptoms (9), Gastrointestinal/surgical (7), Cancer (4), Muscular/connective tissue (3), Blood/immune (2), Other (2), Skin (1), Neuro (1), or Psychiatric/behavioral (1). Thirty-two individuals (19.4%) at the 24-month follow-up required referral to a medical clinic for additional follow-up. All three reinfections with SARS-CoV-2 that required hospitalisation occurred between November 2020 and February 2021, before any vaccine doses were given. Median disease severity of the COVID-19 reinfections that required hospitalisation (n = 3) was WHO CPS 5 (range 4–6), and the median length of hospital stay was 4 days (range 1–20). No patient required invasive mechanical ventilation. With regard to dyspnea (assessed using the mMRC dyspnea scale), the three re-infected individuals reported a median improvement of 2 points (range 0–2) between the 4-month and the 24-month assessments. However, self-rated health deteriorated by a median of 1 point (range −1 to +1) on a five-point Likert scale in the same individuals.

### Symptoms at 24 months

Significant improvements in the degree to which each symptom affected daily life were seen in the entire cohort as well as the ICU and non-ICU subgroups regarding the following symptoms: weakness/fatigability in arms/legs, difficulty being physically active, increased sleep, mental fatigue, and difficulty managing work/studies (Table 2).

**Table 2:** All registered symptoms.

Symptoms	All (n = 165)	p-value	ICU (n = 47)	p-value	Non-ICU (n = 118)	p-value
Domain I—Visual symptoms						
Photophobia	57 (35%) 2 (2–3)	0.13	14 (30%) 2 (2–4)	0.17	43 (37%) 2 (2–3)	0.33
Difficulty or discomfort when altering focus	23 (14%) 3 (2–4)	0.51	8 (17%) 3 (2–3)	0.81	15 (13%) 3 (3–4)	0.53
Blurred vision/double vision	40 (24%) 3 (2–3)	0.41	11 (23%) 3 (2–3)	0.92	29 (25%) 3 (2–3)	0.27
Difficulty reading	45 (27%) 3 (2–4)	0.91	8 (17%) 3 (2–4)	0.96	35 (30%) 3 (2–4)	0.91
Difficulty watching fast moving objects such as TV	29 (18%) 3 (2–3)	0.95	8 (17%) 3 (2–3)	0.75	21 (18%) 3 (2–3)	0.92
Sensitivity to visual motion in busy environments	56 (34%) 3 (2–3)	0.86	17 (36%) 3 (2–4)	0.21	39 (33%) 3 (2–3)	0.34
Headache	51 (31%) 3 (2–4)	0.08	12 (26%) 2 (2–3)	0.34	39 (33%) 3 (2–4)	0.16
Domain II—Sensorimotor symptoms						
Weakness/fatigability in arms/legs	96 (58%) 3 (2–4)	**≤0.001**	28 (60%) 3 (2–3)	**≤0.001**	68 (58%) 3 (2–4)	**0.01**
Difficulty walking >1 km	55 (33%) 3 (3–4)	**0.002**	10 (21%) 3 (3–4)	**0.001**	45 (38%) 3 (3–4)	0.08
Difficulty being physically active	103 (62%) 3 (2–4)	**≤0.001**	28 (60%) 3 (2–4)	**≤0.001**	75 (64%) 3 (3–4)	**0.02**
Muscular soreness/discomfort	93 (56%) 3 (2–3)	0.38	31 (66%) 3 (2–4)	0.71	62 (53%) 3 (2–3)	0.24
Difficulty driving a car/using public transport	22 (13%) 3 (2–4)	0.10	5 (11%) 3 (2–3)	**0.006**	17 (14%) 3 (2–4)	0.96
Altered bodily sensations	50 (30%) 2 (2–3)	0.32	18 (38%) 3 (2–3)	0.05	32 (27%) 2 (2–3)	0.91
Difficulties performing personal hygiene or dressing	22 (13%) 3 (2–4)	0.32	4 (9%) 3 (3–3)	**0.03**	18 (15%) 3 (2–4)	0.81
Domain III—Cognitive symptoms						
Difficulty remembering	99 (60%) 3 (2–4)	0.88	19 (40%) 3 (2–3)	0.17	80 (68%) 3 (2–4)	0.57
Word finding difficulties	80 (48%) 3 (2–3)	0.43	20 (43%) 3 (2–3)	0.40	60 (51%) 3 (2–3)	0.71
Mental Slowness	64 (39%) 3 (2–4)	**0.02**	13 (28%) 3 (2–3)	0.08	51 (43%) 3 (2–4)	0.10
Difficulty multitasking	63 (38%) 3 (2–4)	0.36	15 (32%) 3 (2–3)	0.37	48 (41%) 3 (2–4)	0.65
Difficulty concentrating	78 (47%) 3 (2–4)	**0.01**	16 (34%) 3 (2–3)	**0.05**	62 (53%) 3 (2–4)	0.06
Difficulty expressing thoughts when speaking	62 (38%) 3 (2–3)	0.90	17 (36%) 3 (2–3)	0.96	45 (38%) 3 (2–3)	0.91
Difficulty participating in social activities	64 (39%) 3 (2–3)	0.31	17 (36%) 3 (2–3)	0.80	47 (40%) 3 (3-3)	0.22
Increased sleep (>2 h difference)	17 (10%) 3 (2–3)	**0.004**	1 (2%) 3 (3–3)	**0.03**	16 (14%) 3 (2–3)	**0.03**
Domain IV—Affective symptoms						
Feeling anxious	62 (38%) 3 (2–4)	**0.05**	19 (40%) 3 (2–3)	0.24	43 (36%) 3 (2–4)	0.11
Feeling low/depressed	66 (40%) 3 (2–4)	0.38	18 (38%) 3 (2–4)	0.97	48 (41%) 3 (2–4)	0.30
Domain V—Dysphagia						
Difficulty swallowing	34 (21%) 3 (2–3)	0.30	9 (19%) 2 (1–3)	0.96	25 (21%) 3 (2–3)	0.31
Domain VI—Voice/language abnormalities						
Dysphonia	54 (33%) 3 (2–3)	0.53	11 (23%) 3 (2–3)	0.11	43 (36%) 3 (2–3)	0.89
Dysarthria	25 (15%) 3 (2–3)	0.91	6 (13%) 3 (2–4)	0.32	19 (16%)2 (2–3)	0.52
Difficulty understanding speech	26 (16%) 3 (2–3)	0.22	4 (9%) 2 (2–3)	**0.03**	22 (19%) 3 (2–3)	0.89
Domain VII—Fatigue						
Sleep less (>2 h difference)	44 (27%) 3 (2–4)	0.08	13 (28%)1 (1-1)	0.65	27 (23%)3 (3–4)	0.07
Stress sensitivity/irritability	90 (55%) 3 (2–4)	0.46	27 (57%) 3 (2–4)	0.44	63 (53%) 3 (2–4)	0.19
Phonophobia	65 (39%) 3 (2–4)	0.96	17 (36%) 3 (3-3)	0.87	48 (41%) 3 (2–4)	0.85
Mental fatigue	108 (65%) 3 (2–4)	**≤0.001**	32 (68%) 3 (2–3)	**0.01**	76 (64%) 3 (2–4)	**≤0.001**
Symptoms not included in domains						
Difficulty managing work/studies	35 (32%) 3 (2–4)	**0.001**	8 (17%) 3 (2–4)	**0.02**	27 (23%) 3 (3–4)	**0.02**
Experienced falls after discharge	20 (12%) 3 (2–4)	0.61	7 (15%) 3 (2–4)	0.27	13 (11%) 3 (2–4)	0.89
Hearing deterioration	43 (26%) 3 (2–4)	0.08	11 (23%) 3 (2–4)	**0.03**	32 (27%) 3 (2–4)	0.43
Altered smell/taste	59 (36%) 3 (2–4)	0.09	10 (21%) 2 (1–3)	0.14	49 (42%) 3 (2–4)	0.23
Dizziness	50 (30%) 3 (2–3)	0.20	14 (30%) 2 (2–3)	0.55	36 (31%) 3 (2–3)	0.07

The prevalence of symptoms is presented as n (%). The degree to which the symptoms affected daily life was graded from 1 to 5 (1: no impact; 2: minor impact; 3: moderate impact; 4: high impact; 5: very high impact) and is presented as median (IQR). The p-values refer to paired comparisons made between the 4-month and 24-month follow-up. All significant findings are in bold and were due to improvements at the individual level from the 4-month follow-up.

**Fig. 2:**
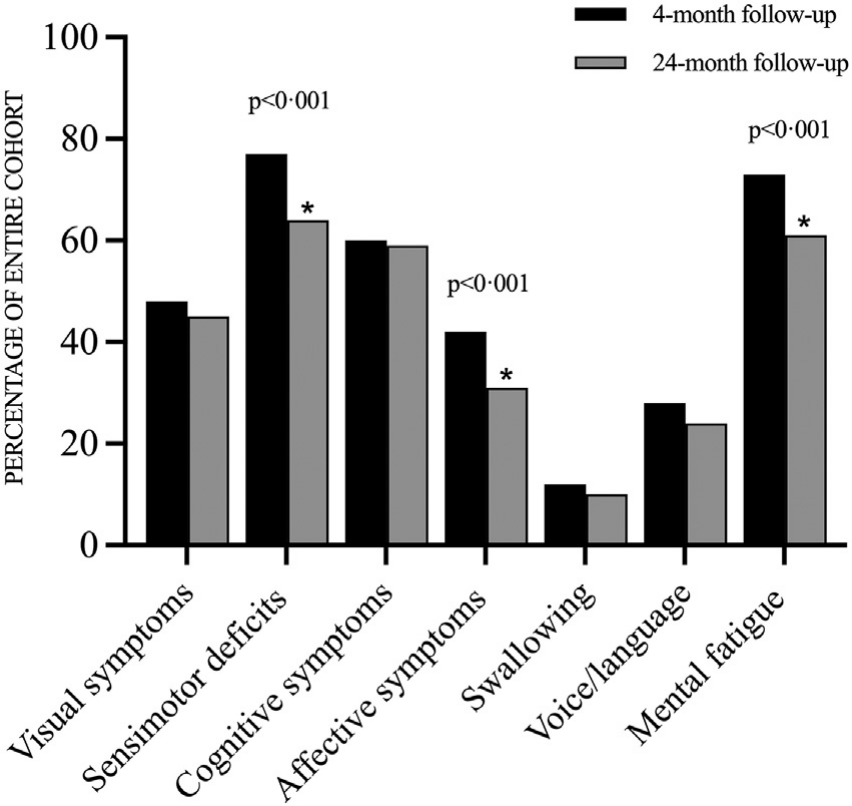
**Symptoms affecting everyday life.** Percentages of participants reporting at least one persisting symptom in the respective domain rated as at least 3/5 (a moderate degree of impact on everyday life).♦Significant improvement at 24 months (p < 0.001) according to the paired Wilcoxon signed-rank test.

A total of 84.2% (139/165) reported at least one residual symptom posing at least a moderate impact on daily life (degree 3/5 or worse) at the 24-month follow-up (Fig. 2). At the domain level, significant improvements compared with the 4-month follow-up were seen for domains II (sensorimotor deficits), IV (affective symptoms) and VII (mental fatigue). For the ICU-treated subgroup, the only significant improvement at the domain level was for domain II (sensorimotor deficits).

The prevalence of symptoms with at least a moderate impact on daily life had decreased significantly at the 24-month assessment compared to the 4-month assessment for the following symptoms: weakness/fatigability in arms/legs, difficulty walking at least 1 km, difficulty being physically active, difficulty managing work/studies, increased need for sleep, headache, mental fatigue, and anxiety. For a detailed presentation of all symptoms graded 3/5 or worse at four and 24 months respectively, see Appendix Table S1. Similar improvements were seen when the data were compared over time at an ordinal level (Table 2). Thirty-one individuals (18.8%) reported additional symptoms not included in the 37 specific questions of the interview, such as palpitations, chronic fever, reduced appetite, sweating, and hair loss.

**Fig. 3:**
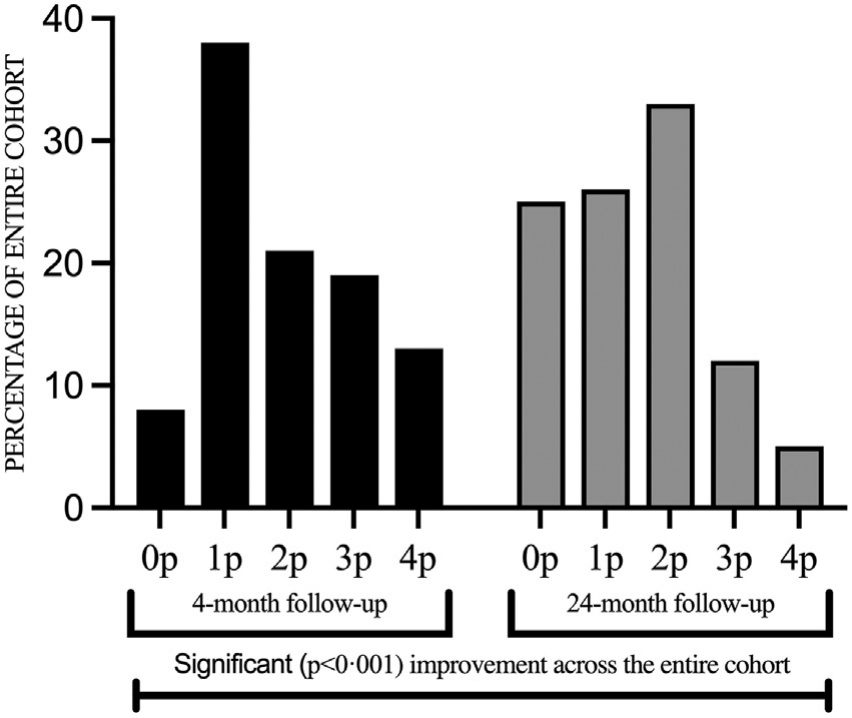
**Breathlessness—mMRC dyspnea scale.** Perceived dyspnea as assessed by the modified Medical Research Council (mMRC) dyspnea scale, comparing the 4-month (black) and 24-month (grey) follow-ups. 0p: “I only get breathless with strenuous exercise”, 1p: “I get short of breath when hurrying on level ground or walking up a slight hill”, 2p: “I walk slower than people of the same age on level ground because of breathlessness or have to stop for breath when walking at my own pace on level ground”, 3p: “I stop for breath after walking about 100 yards or after a few minutes on level ground”, 4p: “I am too breathless to leave the house” or “I become breathless when dressing”. Using the paired Wilcoxon signed-rank test a significant improvement was seen for the entire cohort at 24 months (p < 0.001).

The degree of breathlessness as assessed using paired individual comparisons on the mMRC scale improved significantly (p < 0.001) between the 4- and 24-month follow-ups (Fig. 3), for an alluvial diagram, see Supplementary Fig. S3. The number of individuals who were asymptomatic (mMRC = 0) increased from 14 (8%) at the initial 4-month assessment to 41 (25%) at the 24-month follow-up. The proportion of individuals with light (mMRC = 1) or moderate (mMRC = 2) dyspnea was similar at the 4-month and the 24-month assessments, 98/164 (60%) and 95/165 (58%), respectively. Regarding severe dyspnea (mMRC grade 3 or 4), the proportion decreased from 32% (52/164) to 18% (29/165) between the assessments. At the 24-month follow-up, the median score on the mMRC scale for the entire cohort was 2 (IQR 1–2), for ICU-treated patients 1 (0–2) and for non-ICU treated patients 2 (1–2).

### Occupational status

**Supplementary Fig. S1:**
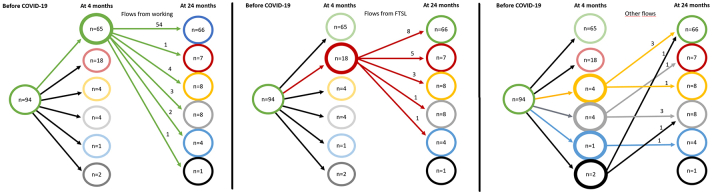
Occupational status flow-chart. Alluvial diagram of occupational status before COVID-19, at four months post-discharge and at 24 months post-admission for individuals who were working or studying (n = 94) before COVID-19. All three images represent the same picture, with some parts highlighted in each for clarity. The numbers in each circle indicate the total number of individuals with a particular occupational status at a given point in time. The coloured lines and their corresponding number represent the flows of individuals from the respective groups (e.g., red for FTSL) based on 4-month occupational status. Green = Working or studying. Blue = Retired. Grey = Unemployed. Yellow = Part-time sick leave (PTSL). Red = Full-time sick leave (FTSL). Black = Missing data/unknown. Panels a), b) and c) detail the flows of individuals from the 4-month assessment to the 24-month assessment. Panel a (left) shows the change in occupational status from the 4-month to the 24-month follow-ups specifically for those individuals who were still *working or studying* at the 4-month follow-up. Panel b (middle) shows the change in occupational status for those who were working or studying before COVID-19 but *were on FTSL* at the 4-month follow-up. Panel c (right) shows the change in occupational status for those who were working or studying before COVID-19 but were *not working/studying or on FTSL at 4 months* post-discharge (i.e., 4-month occupational status was PTSL, unemployed, retired or unknown). The right-most column in each panel shows the *total* number of individuals in each category at the 24-month follow-up (and is therefore identical in all three panels).

Of the 165 participants in the entire cohort, 94 (57%) were working or studying prior to COVID-19. Supplementary Fig. S1 shows a detailed analysis of changes in occupational status at an individual level for these 94 individuals. At the 4-month assessment, 65 (69%) of those patients were still working or studying (Supplementary Fig. S1, panel a), four (4%) were unemployed, 18 (19%) were on full-time sick leave (FTSL), four (4%) were on part-time sick leave (PTSL), one (1%) had retired and data was missing for two individuals (2%). For the 18 individuals that were not on sick leave before COVID-19, but were on FTSL at the 4-month follow-up (Supplementary Fig. S1, panel b), 8 (44%) had returned to work or studying at the 24-month follow-up, 3 (17%) were on PTSL, one (6%) had retired and one (6%) was unemployed.

The number of individuals in the entire cohort who were employed or studying was significantly lower at 24 months compared with before COVID-19 (p < 0.0001).

### Self-rated health

**Fig. 4:**
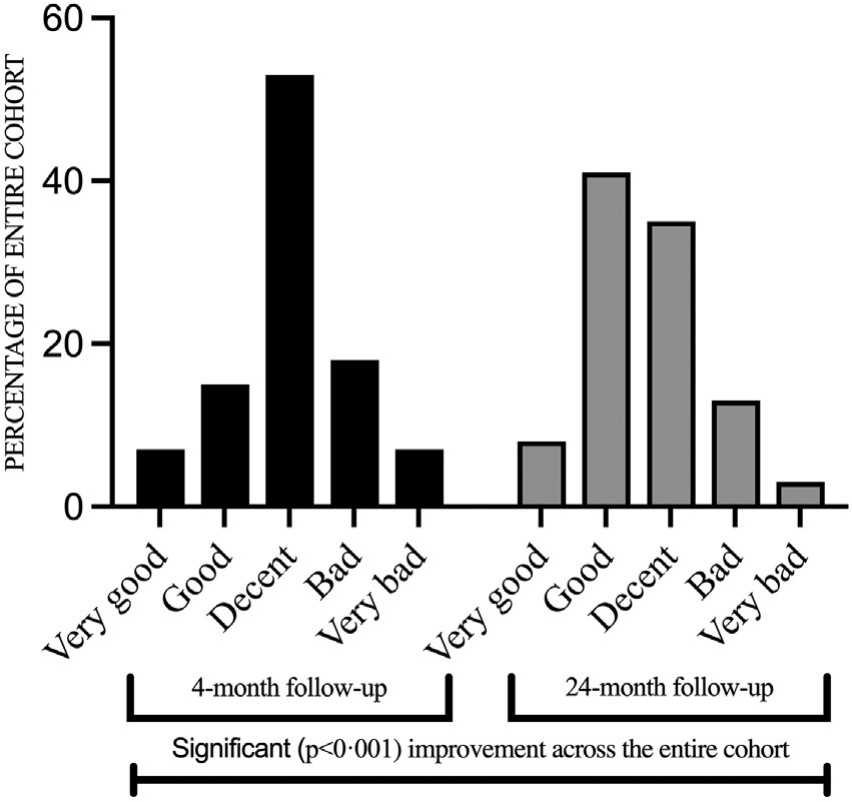
**Self-rated health.** Self-rated general health on a 5-point Likert scale from very good to very bad at the 4 and 24-month follow-ups, respectively. Using the paired Wilcoxon signed-rank test a significant improvement was seen for the entire cohort at 24 months (p < 0.001).

**Supplementary Fig. S2:**
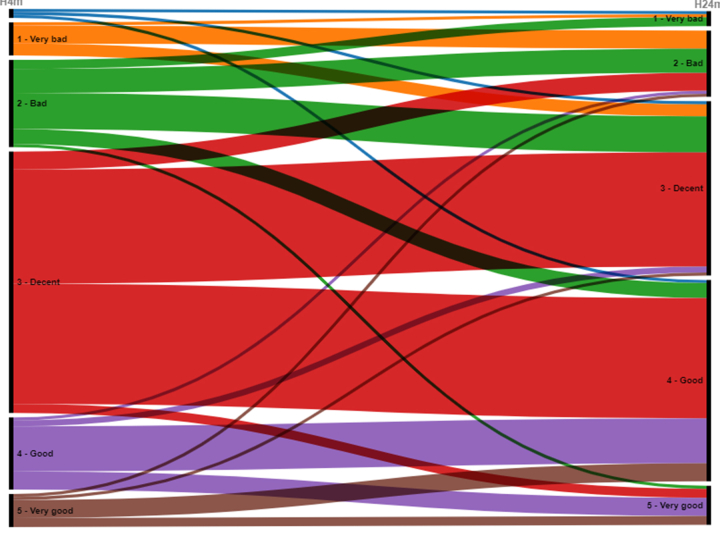
Alluvial diagram of self-rated health. Self-rated general health on a 5-point Likert scale from very good to very bad at the 4 and 24-month follow-ups, respectively. Using the paired Wilcoxon signed-rank test a significant improvement was seen for the entire cohort at 24 months (p < 0.001).

**Supplementary Fig. S3:**
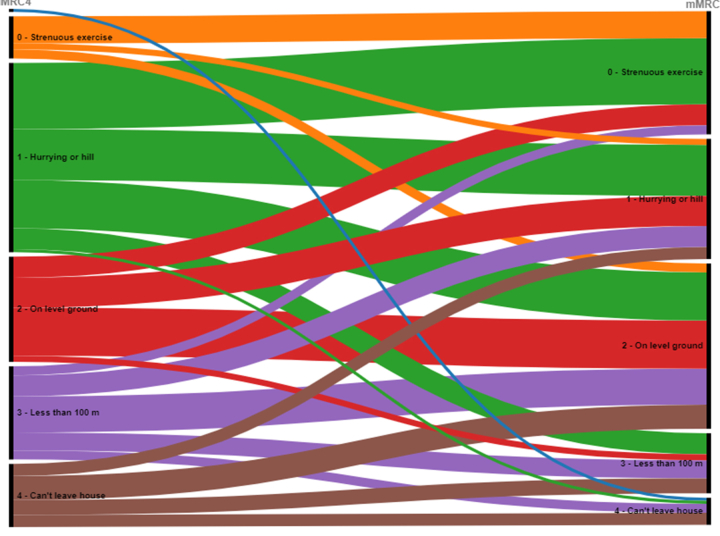
Alluvial diagram of breathlessness assessed on the mMRC scale. Perceived dyspnea as assessed by the modified Medical Research Council (mMRC) dyspnea scale, comparing the 4-month (left-hand side) and 24-month (right-hand side) follow-ups. 0p: “I only get breathless with strenuous exercise”, 1p: “I get short of breath when hurrying on level ground or walking up a slight hill”, 2p: “I walk slower than people of the same age on level ground because of breathlessness or have to stop for breath when walking at my own pace on level ground”, 3p: “I stop for breath after walking about 100 yards or after a few minutes on level ground”, 4p: “I am too breathless to leave the house” or “I become breathless when dressing”. Using the paired Wilcoxon signed-rank test a significant improvement was seen for the entire cohort at 24 months (p < 0.001).

Self-rated general health significantly improved (p < 0.001) between the 4 and 24-month follow-ups (Fig. 4). The number of individuals who rated their general health as good or very good increased from 35 individuals (22%) at the 4-month assessment to 80 (49%) at the 24-month assessment. The median score for self-rated health at the 24-month follow-up for the entire cohort was 3 (2–3), for ICU-treated patients 2 (2–3) and for non-ICU-treated patients 3 (2–3). A sub-group analysis was performed, comparing unvaccinated vs. fully vaccinated individuals (those who had received three or more doses at the time of the 2-year follow-up). It revealed a significant difference (p = 0.002) between the groups, where the fully vaccinated group (n = 122) rated their health as having improved while the unvaccinated group (n = 10) rated their health as having deteriorated between the 4- and 24-month assessments. Regarding the subgroup of participants with psychiatric comorbidity at baseline (n = 18), self-rated health improved similarly to the rest of the cohort (n = 144), p = 0.88. An alluvial diagram is also available (Supplementary Fig. S2).

## Discussion

To the best of our knowledge, this is the first study to report 2-year outcomes in patients with PCC previously hospitalised due to COVID-19 in a European country. Over 80% of patients with PCC at four months after hospitalisation still experienced symptoms and activity/participation limitations affecting everyday life at the 2-year follow-up. Based on Fig. 1, this means that at least 30% (139/460) of survivors at four months after hospitalisation for COVID-19 still experienced symptoms affecting everyday life after two years. The most common symptoms at 24 months post-admission were related to cognition, sensorimotor function, and mental fatigue. Significant improvements from the 4 to 24-month follow-up were however seen for general health, dyspnea, sensorimotor complaints, cognitive symptoms, affective symptoms, and mental fatigue.

The persisting symptoms reported in the current study at 24 months post-hospital admission correspond to those previously reported in a meta-analysis on individuals with PCC up to 12 months post-hospital discharge.^27^ In a Dutch study,^28^ 92% reported at least one residual symptom still present at 12 months after hospitalisation for COVID-19. Similar to our study, they reported significant improvements in muscle weakness and exertional dyspnea from three to 12 months, but contrastingly not in fatigue or cognitive symptoms.^28^ Similar results to ours were also reported in a longitudinal Spanish study exploring symptom trajectories,^29^ in which 81% of individuals had at least one symptom still present at eight months after hospital admission, with this number decreasing to 68% at 13 months. In a study from Verona, Italy,^30^ where 51% of participants were hospitalised for COVID-19 and the rest were managed in outpatient care, 20% reported at least one symptom remaining at nine months. The lower percentage compared with the 30% observed in our study may be explained by specific differences between the cohorts. Firstly, only half of the Italian cohort was hospitalised (of which 10% were admitted to the ICU), indicating less severe disease than in our cohort. Secondly, 98% of the Italian cohort reported their pre-COVID-19 health status as very good or excellent, compared with 72% of our cohort (assessed on a similar 5-point Likert scale). Additionally, in line with the present study, a Chinese 2-year follow-up of initially hospitalised patients identified overall improvements regarding both physical and mental health but with a high symptom load remaining 2 years after infection.^31^

It has previously been demonstrated that significant cognitive deficits can persist post-COVID-19 even in individuals that do not report such symptoms.^11^^,^^32^ This suggests that the self-reported prevalence of cognitive symptoms in our study might underestimate the true prevalence of such deficits. Additionally, earlier experiences from SARS and MERS infections portray long-term persistent neuropsychiatric deficits that are not necessarily correlated to initial clinical severity,^33^, ^34^, ^35^ which is in many ways similar to PCC and the results of the present study.

Lock-downs enforced to limit the spread of infection may not only increase the risk of depression and anxiety disorders,^36^ but could also impair cognitive function.^37^^,^^38^ Sweden had few restrictions and lock-down procedures as compared to other countries, described in detail by a commission designated by the Swedish government.^39^ Large public gatherings of more than 50 individuals were at times prohibited. No strict lock-down was enforced. The Swedish government also issued economic support to many companies which enabled continued employment and continued work, albeit from a distance when possible. The Public Health Agency of Sweden also recommended that individuals aged 70 or older, as well as groups at high risk for severe COVID-19, take precautions. Kivi and colleagues presented that during the initial pandemic wave Swedish older adults rated their general well-being at a similar level to, or even higher than, before the pandemic.^40^ In summary, our results may to some extent be explained as a possible consequence to pandemic-related restrictions and social distancing, as we have no control group to compare with, but we do not believe it to play a decisive part.

A surprising finding of the current study was the tendency towards lower degrees of residual symptoms among the ICU-treated subgroup, of which a majority (91%) received invasive mechanical ventilation, compared with the non-ICU-treated subgroup. The greater predominance of males in the ICU subgroup (81%) compared with the non-ICU subgroup (56%) may have influenced this observation, as female sex has been proposed as a risk factor for residual symptoms of PCC at least 12 months after infection.^27^ Furthermore, survivors from intensive care due to other diagnoses often experience long-lasting residual symptoms, such as neurocognitive, affective and pulmonary symptoms, as well as activity impairments.^41^ Many of these symptoms are in line with those presented in our study. It is therefore challenging to determine which symptoms, if any, are specifically related to COVID-19 and which are more general post-ICU symptoms. However, as the prevalence of residual symptoms was similar in the non-ICU-treated subgroup, we suspect that the residual symptoms reported in this study cannot be solely explained as a post-ICU phenomenon.

It has been hypothesised that endothelial dysfunction due to COVID-19 may be a contributing factor to long-lasting symptoms in PCC,^42^^,^^43^ and the most common causes for hospital admission in the interim period of the present study were indeed cardiovascular. This is in line with a recently published large cohort study by Wang and colleagues,^44^ presenting a higher incidence of cardiovascular disease twelve months after initial SARS-CoV-2 infection. Hospital readmission rates after COVID-19 vary between countries. A meta-analysis by Ramzi revealed an all-cause one-year readmission rate of 10.7% in developed countries.^45^ The presented 2-year readmission rate of 21.2% in our cohort may suggest that patients with PCC are at higher risk for hospital readmissions. However, since the readmission rate was not registered for the entire hospitalised cohort (including those without PCC) such a comparison is beyond the scope of this article.

More than half of the patients in the current study who were actively working/studying before COVID-19, but were on FTSL at the initial 4-month follow-up, had returned to either part or full-time work at 24 months. Occupational status was however still significantly worse at the 2-year follow-up compared to pre-COVID. Prior studies suggest that rates of sick leave after COVID-19 tend to decrease or even normalise to that of the general population within four to five months of disease onset.^46^, ^47^, ^48^ Our results show that rates of sick leave in previously hospitalised patients with PCC may decrease but remain high two years later.

Vaccination before SARS-CoV-2 infection may reduce the risk of developing PCC,^49^ but whether vaccination post-infection ameliorates already established residual symptoms is still unclear, with some studies showing a reduction in symptoms and others no effect or even worsening of symptoms.^49^ No effective vaccines were available before infection for the current cohort, but after infection a majority had received at least three doses at the 24-month follow-up. As such, individual vaccinations may have influenced our results in either way. However, our results suggest that vaccination after development of PCC leads to an improvement in self-rated health as compared to being unvaccinated.

All patients in our study were offered a clinical follow-up by a multi-professional rehabilitation team, which the majority (158, 85%) attended. Despite this, symptoms affecting everyday life remained at the 24-month follow-up. There is a potential risk for an even higher number of remaining symptoms in patients that are not offered the same medical follow-up. In the living guideline for clinical management of COVID-19 updated by the WHO on the 15th of September 2022,^50^ several rehabilitation services for PCC are recommended, e.g., multidisciplinary rehabilitation teams, follow-up and referral systems, standardised symptom assessments, education and skills training regarding energy conservation routines. Many of these recommendations are in line with the clinical follow-up offered to our cohort at the 4-month assessment. Our results strengthen these recommendations, as there seems to be a need for medical attention in patients with PCC even two years after initial infection, with potential for significant improvement of many symptoms. However, it also indicates a need for further studies of specific interventions and their effectiveness in long-term rehabilitation of patients with PCC.

### Strengths and limitations

Strengths of the present study include the long-term follow-up of a well-defined cohort as well as a high participation rate (91%), limiting information bias due to loss to follow-up. All interviews were conducted via telephone by medical professionals, which enhances data quality compared with a survey completed independently. Patients were interviewed both at four and at 24 months, thus making prognostic trajectories possible. Additionally, medical records were screened for health issues occurring in the interim period (including reinfections with SARS-CoV-2).

Limitations include the lack of a control group as well as the self-reported format from a subgroup of patients that initially reported lingering symptoms. This may have resulted in a selection bias, by including individuals who were more likely to report symptoms than the general population. A strength of the study that counterbalances this to some extent is that the same individuals were interviewed at both points in time. Additionally, no further evaluations of these self-reported symptoms, such as measurements of lung function, were conducted. As two years had elapsed since hospitalisation, other factors than the initial SARS-CoV-2 infection may have influenced the outcomes. As this cohort represents individuals from the initial pandemic wave, whether the results are applicable in the context of current viral mutations and vaccination statuses before infection is uncertain. It should also be noted that the same structured interview protocol from the 4-month follow-up was used for the 24-month follow-up and thus some symptoms that we now know constitute part of PCC (e.g., postural tachycardia and other dysautonomic symptoms) were omitted. Also, information bias may arise from the fact that the interview protocol is not strictly validated for the study population. However, we have previously reported that a multi-disciplinary clinical assessment attended by the vast majority of the LinCoS cohort also included in this study corroborated self-reported symptoms from the 4-month telephone interview,^11^ which is an indication of low risk of information bias. Furthermore, p-values should be interpreted with caution as potential confounders were not adjusted for, and no correction was made for multiple testing. Lastly, for some sub-analyses the sample size was low.

### Conclusions

Our cohort of patients, who were hospitalised with COVID-19 during the first pandemic wave and showed symptoms indicating PCC at 4-months post-discharge, showed improved symptoms at two years post-admission, but also a high prevalence of persistent cognitive, sensorimotor and fatigue symptoms impacting on their everyday life. This implies a need to establish routines for long-term follow-up of patients previously hospitalised due to COVID-19 with PCC.

## Contributors

All authors contributed to study design. CW, GF and CFE conducted the telephone interviews. JA helped with initial guidance in interview technique. Primarily, CW and GF reviewed the data with assistance from RL, AD, KN, ÅÖB and SB. CW performed most statistical analysis with support from GF who also assisted in validation of the database. RL and AD applied for funding. CW and GF wrote the initial draft and together with RL, AD, KN, ÅÖB and SB edited following versions until submission of the manuscript.

## Data sharing statement

Additional follow-up studies related to the LinCoS project are planned and ongoing. After completion of LinCoS, data can be made available upon request and assessment by the LinCoS project group and will then be provided in a de-identified manner.

## Declaration of interests

The authors declare no conflicts of interest.
