# Supplementary material for: Two-year follow-up of patients with post-COVID-19 condition in Sweden: a prospective cohort study
Source: Lancet Reg Health Eur. 2023 Feb 24;28:100595. doi: 10.1016/j.lanepe.2023.100595 (PMC9951394; doi:10.1016/j.lanepe.2023.100595)
Supplement: Supplementary Table S1 [file mmc1.docx]

**Table S1. Symptoms affecting daily life at least moderately.** Group level point prevalence of symptoms at the 4 and 24-month follow-ups**.** The prevalence of symptoms is presented as n (%). The degree to which the symptoms affected daily life was graded from 3-5 (3: to a moderate degree; 4: to a high degree; 5: to a very high degree) and is presented as median (IQR). The p-values refer to paired comparisons (McNemar’s test) made between the 4 and 24-month follow-ups within each group (i.e., the whole cohort, only ICU, and only non-ICU). P-values < 0.05 are shown in bold. All significant findings were due to improvements at the individual level from the 4-month follow-up.

| **Symptoms – n (%)** | **Total**  **(n=165)** | | **Non-ICU**  **(n=118)** | | **ICU**  **(n=47)** | |
| --- | --- | --- | --- | --- | --- | --- |
|  | **4 months** | **24 months** | **4 months** | **24 months** | **4 months** | **24 months** |
| Domain I - Visual symptoms | | |  |  |  |  |
| Photophobia | 25 (16%)  p=1·00 | 27 (17%) | 20 (18%)  p=1·00 | 21 (18%) | 5 (11%)  p=1·00 | 6 (13%) |
| Difficulty or discomfort when altering focus | 14 (9%) p=1·00 | 15 (9%) | 10 (9%)  p=1·00 | 11 (10%) | 4 (9%)  p=1·00 | 4 (9%) |
| Blurred vision/double vision | 31 (19%)  p=0·35 | 26 (16%) | 21 (21%)  p=0·26 | 19 (16%) | 7 (16%)  p=1·00 | 7 (15%) |
| Difficulty reading | 26 (16%)  p=1·00 | 27 (17%) | 21 (19%)  p=1·00 | 22 (19%) | 5 (11%)  p=1·00 | 5 (11%) |
| Difficulty watching fast moving objects such as TV | 19 (12%) p=1·00 | 20 (12%) | 12 (12%)  p=1·00 | 15 (13%) | 5 (11%)  p=1·00 | 5 (11%) |
| Sensitivity to visual motion in busy environments | 38 (23%) p=0·87 | 36 (22%) | 29 (26%)  p=0·70 | 26 (23%) | 9 (20%)  p=1·00 | 10 (21%) |
| Headache | **45 (27%) p=0·037** | **30 (18%)** | 34 (29%)  p=0·18 | 25 (22%) | 11 (24%)  p=0·11 | 5 (11%) |
| Domain II - Sensorimotor symptoms | | | |  |  |  |
| Weakness/fatigability in arms/legs | **95 (58%) p<0·001** | **67 (41%)** | **64 (59%)**  **p=0·015** | **49 (43%)** | **31 (71%)**  **p<0·001** | **18 (38%)** |
| Difficulty walking >1km | **60 (38%) p=0·003** | **43 (26%)** | 40 (38%)  p=0·08 | 34 (29%) | **20 (44%)**  **p=0·007** | **9 (19%)** |
| Difficulty being physically active | **104 (63%)**  **P<0·001** | **75 (45%)** | **74 (66%)**  **p=0·012** | **57 (49%)** | **30 (67%)**  **p=0·001** | **18 (38%)** |
| Muscular soreness/discomfort | 61 (37%)  p=0·451 | 57 (35%) | 44 (39%  p=0·31 | 38 (33%) | 17 (39%)  p=1·00 | 19 (40%) |
| Difficulty driving a car/using public transport | 20 (14%) p=0·078 | 13 (8%) | 11 (11%)  p=0·79 | 10 (9%) | **9 (21%)**  **p=0·016** | **3 (6%)** |
| Altered bodily sensations | 25 (15%) p=0·33 | 21 (13%) | 12 (11%)  p=0·80 | 11 (10%) | 13 (30%)  p=0·34 | 10 (22%) |
| Difficulties performing personal hygiene or dressing | 16 (10%) p=0·65 | 14 (8%) | 9 (8%)  p=1·00 | 11 (9%) | 7 (16%)  p=0·22 | 3 (6%) |
| Domain III - Cognitive symptoms | | | |  |  |  |
| Difficulty remembering | 67 (41%) p=0·65 | 65 (40%) | 52 (46%)  p=1·00 | 54 (47%) | 15 (34%)  p=0·34 | 11 (24%) |
| Word finding difficulties | 53 (33%) p=0·11 | 44 (27%) | 39 (35%)  p=0·35 | 34 (29%) | 14 (32%)  p=0·23 | 10 (22%) |
| Mental Slowness | 56 (34%) p=0·080 | 43 (26%) | 44 (39%)  p=0·26 | 36 (31%) | 12 (27%)  p=0·18 | 7 (15%) |
| Difficulty multitasking | 53 (32%) p=0·10 | 42 (26%) | 40 (35%)  p=0·33 | 34 (29%) | 13 (30%)  p=0·23 | 8 (17%) |
| Difficulty concentrating | **68 (42%) p=0·007** | **50 (31%)** | 41 (46%)  p=0·055 | 39 (34%) | 17 (39%)  p=0·065 | 11 (23%) |
| Difficulty expressing thoughts when speaking | 38 (24%) p=0·33 | 33 (20%) | 27 (25%)  p=0·45 | 24 (21%) | 11 (24%)  p=0·75 | 9 (20%) |
| Difficulty participating in social activities | 39 (25%) p=0·74 | 46 (28%) | 28 (25%)  p=0·35 | 37 (31%) | 11 (24%)  p=0·51 | 9 (20%) |
| Increased sleep (>2h difference) | **24 (15%)  p=0·004** | **9 (5%)** | **19 (18%)**  **p=0·019** | **8 (7%)** | 5 (11%)  p=0·22 | 1 (2%) |
| Domain IV - Affective symptoms | | | |  |  |  |
| Feeling anxious | **57 (35%)**  **p=0·029** | **40 (25%)** | 43 (38%)  p=0·072 | 29 (26%) | 14 (33%)  p=0·34 | 11 (23%) |
| Feeling low/depressed | 53 (33%) p=0·18 | 40 (25%) | 41 (36%)  p=0·25 | 32 (28%) | 12 (27%)  p=0·63 | 9 (20%) |
| Domain V - Dysphagia | |  |  |  |  |  |
| Difficulty swallowing | 19 (13%) p=0·33 | 17 (10%) | 14 (15%)  p=0·42 | 13 (11%) | 5 (12%)  p=1·00 | 4 (9%) |
| Domain VI - Voice/language abnormalities | | |  |  |  |  |
| Dysphonia | 32 (21%) p=0·31 | 29 (18%) | 24 (23%)  p=0·45 | 22 (19%) | 8 (18%)  p=0·73 | 7 (15%) |
| Dysarthria | 11 (7%) p=1·00 | 13 (8%) | 9 (9%)  p=0·75 | 9 (8%) | 2 (4%)  p=0·63 | 4 (9%) |
| Difficulty understanding speech | 20 (13%) p=0·38 | 15 (9%) | 14 (13%)  p=1·00 | 14 (12%) | 6 (13%)  p=0·063 | 1 (2%) |
| Domain VII - Fatigue | |  |  |  |  |  |
| Sleep less (>2h difference) | 39 (24%) p=0·067 | 28 (17%) | 28 (25%)  p=0·078 | 20 (18%) | 11 (24%)  p=0·75 | 8 (18%) |
| Stress sensitivity/irritability | 69 (44%) p=0·33 | 64 (39%) | 53 (49%)  p=0·13 | 46 (40%) | 16 (36%)  p=0·80 | 18 (39%) |
| Phonophobia | 43 (27%) p=0·87 | 44 (27%) | 33 (30%)  p=0·57 | 31 (27%) | 10 (23%)  p=0·77 | 13 (28%) |
| Mental fatigue | **111 (69%) p<0·001** | **67 (41%)** | **85 (78%)**  **P<0·001** | **52 (46%)** | **26 (58%)**  **p=0·013** | **16 (35%)** |
| Symptoms not included in domains | | | |  |  |  |
| Difficulty managing work/studies | **35 (43%) p=0·007** | **24 (22%)** | 25 (40%)  p=0·077 | 20 (25%) | 10 (53%)  p=0·070 | 5 (19%) |
| Experienced falls after discharge | 12 (7%) p=1·00 | 12 (7%) | 9 (8%)  p=1·00 | 8 (7%) | 3 (7%)  p=1·00 | 4 (9%) |
| Hearing deterioration | 21 (13%) p=0·44 | 26 (16%) | 19 (17%)  p=1·00 | 20 (17%) | 2 (4%)  p=0·22 | 6 (13%) |
| Altered smell/taste | 34 (22%) p=0·33 | 30 (18%) | 30 (26%)  p=0·38 | 27 (23%) | 4 (9%)  p=1·00 | 4 (9%) |
| Dizziness | 37 (23%)  p=0·14 | 28 (17%) | 32 (28%)  p=0·10 | 23 (20%) | 5 (11%)  p=1·00 | 5 (11%) |
