# Supplementary material for: Two-year follow-up of patients with post-COVID-19 condition in Sweden: a prospective cohort study
Source: Lancet Reg Health Eur. 2023 Feb 24;28:100595. doi: 10.1016/j.lanepe.2023.100595 (PMC9951394; doi:10.1016/j.lanepe.2023.100595)
Supplement: Supplemental Material [file mmc2.docx]

**Interview questions**

**Section A**

Consider the following question for each of the items below.

“At this moment in time, do you experience that the issue below comprises a new or aggravated problem as compared to prior to COVID-19 infection?”

If yes, to what extent does this new or aggravated problem affect your daily life?

1: no impact; 2: to a minor degree; 3: to some degree; 4: to a high degree; 5: to a very high degree

·       Weakness/fatigability in arms and/or legs

·       Difficulty walking >1km

·       Difficulty being physically active

·       Experienced falls post-discharge

·       Altered bodily sensation

·       Muscular soreness/ aches/ cramps/ discomfort

·       Difficulty swallowing

·       Altered smell and/ or taste

·       Difficulty hearing

·       Blurred vision/double vision

·       Difficulty watching fast moving objects such as TV

·       Difficulty or discomfort when altering focus or gaze

·       Increased light sensitivity (photophobia)

·       Increased sound sensitivity (phonophobia)

·       Headache

·       Dizziness

·       Sleep less/ disturbed sleep (>2 hours change)

·       Increased need for sleep (>2 hours change)

·       Mental fatigue/ fatigability

·       Stress sensitivity/ irritability

·       Feeling anxious

·       Feeling low/ depressed

·       Difficulty concentrating

·       Difficulty multi-tasking

·       Mental slowness

·       Difficulty remembering

·       Difficulty understanding speech

·       Difficulty word-finding when speaking

·       Difficulty expressing thoughts when speaking

·       Slurred/ indistinct speech (dysarthria)

·       Weak/ hoarse voice (dysphonia)

·       Sensitivity to visual motion in busy environments

·       Difficulty participating in social activities (socialising with family and friends)

·       Difficulty managing work/ studies

·       Difficulty driving a car/ using public transport*****

·       Difficulty performing personal hygiene and dressing

·       Difficulty reading

**Section B**

modified Medical Research Council (mMRC) Dyspnea Scale

0 - “I only get breathless with strenuous exercise”

1 - “I get short of breath when hurrying on the level or walking up a slight hill”

2 - “I walk slower than people of the same age on the level because of breathlessness or have to stop for breath when walking at my own pace on the level”

3 - “I stop for breath after walking about 100 yards or after a few minutes on the level”

4 - “I am too breathless to leave the house” or “I am breathless when dressing”

**Section C**

At the moment, would you say your health is:

Very good; Good; Decent; Bad; Very bad
